# Fractal measures of spatial pattern as a heuristic for return rate in vegetative systems

**DOI:** 10.1098/rsos.150519

**Published:** 2016-03-30

**Authors:** M. A. Irvine, E. L. Jackson, E. J. Kenyon, K. J. Cook, M. J. Keeling, J. C. Bull

**Affiliations:** 1 Centre for Complexity Science, Zeeman Building, University of Warwick, Coventry CV4 7AL, UK; 2 School of Medical and Applied Sciences, Central Queensland University, North Rockhampton, Queensland, Australia; 3 School of Life Sciences, University of Sussex, Brighton, UK; 4 Natural England, Truro, UK; 5 Mathematics Institute and Department of Biological Sciences, University of Warwick, Gibbet Hill Road, Coventry CV4 7AL, UK; 6 Department of Biosciences, University of Swansea, Swansea, UK

**Keywords:** return rate, fractal growth, self-organization, persistence, ecological indicators, Korcak exponent

## Abstract

Measurement of population persistence is a long-standing problem in ecology; in particular, whether it is possible to gain insights into persistence without long time-series. Fractal measurements of spatial patterns, such as the Korcak exponent or boundary dimension, have been proposed as indicators of the persistence of underlying dynamics. Here we explore under what conditions a predictive relationship between fractal measures and persistence exists. We combine theoretical arguments with an aerial snapshot and time series from a long-term study of seagrass. For this form of vegetative growth, we find that the expected relationship between the Korcak exponent and persistence is evident at survey sites where the population return rate can be measured. This highlights a limitation of the use of power-law patch-size distributions and other indicators based on spatial snapshots. Moreover, our numeric simulations show that for a single species and a range of environmental conditions that the Korcak–persistence relationship provides a link between temporal dynamics and spatial pattern; however, this relationship is specific to demographic factors, so we cannot use this methodology to compare between species.

## 1. Introduction

The description and prediction of spatial patterns in nature has fascinated theoreticians and applied ecologists alike for many years. Initially, the study of seemingly intractable, complex patterns in time and space was predominantly a descriptive science [1]. However, in the latter years of the previous century, theoretical advancements demonstrated that complex spatio-temporal patterns could be generated as emergent properties resulting from simple rules [2]. This opened the door to understanding the mechanistic basis of many types of ecological pattern, including regular [3] and fractal/scale-free geometries [4]. However, the inverse problem of inferring biological mechanisms from observations and measurements on ecological systems remains a key challenge [5–8]. In part this is because, surprisingly, very similar spatial patterns can arise from a wide range of environmental and underlying endogenous processes [9–11]. In this study, we combine theoretical analysis of a simple but generally applicable, spatio-temporal simulation model with statistical modelling of independent spatial and temporal datasets from a well-studied ecosystem: a temperate seagrass monoculture. We explore the theoretical and empirical relationships between spatial and temporal measures of underlying dynamics, as well as providing some discussion of the potential, and limitations, of using spatial metrics in order to quantify ecological dynamics.

Broadly speaking, a spatio-temporal system (such as a spatial vegetative system) will typically have a high number of dimensions and attempting to match model and data by comparing the precise locations of individuals would quickly become intractable for all but trivial system sizes. This introduces the idea of using summary statistics to encapsulate the key information pertaining to the underlying dynamics of a given ecological growth process [12]. Traditionally, these techniques have taken the form of statistics derived from longitudinal measures of the total population size, ignoring spatial structure [13–15]. One important measure of resilience is the return rate or engineering resilience [16,17]. Given a stable ecosystem, this measures how long on average it takes before the system returns to the stable equilibrium point following a disturbance. The return rate quantifies this, and can be taken mathematically as the dominant eigenvalue of the dynamic system around the stable equilibrium value. However, often in order to gain accurate statistics many sequential observations need to be taken over extended periods of time. By contrast, spatial statistics derived from remote sensing techniques have a high number of degrees of freedom: they can be produced rapidly, can generate large amounts of data and therefore, in theory may lead to insights similar to those from more classical long-term studies [2,18,19].

The theory of fractals and scaling has numerous applications in ecology and is intimately linked with the ideas of spatial dynamics and inferring process from pattern. The original theory, proposed by Mandelbrot, was used to explain certain seemingly ubiquitous patterns in nature [20]. Since this time there has been much tantalizing speculation over using fractal theory to elucidate ecologically meaningful parameters from spatial patterns.

A major application of fractal theory in ecology is in the scaling of vegetation patch sizes. The distribution can be described by a power law in certain settings such as semi-arid ecosystems [21]. The distribution also tends to develop an increasing truncated tail as the system moves closer to a critical threshold due to increasing environmental pressure [22]. This implies that a spatial snapshot of a vegetation distribution can be used to determine if an ecosystem is close to a threshold that would lead to ecosystem collapse. The truncation of the power law may not universally be an indicator of ecosystem collapse, where the simpler measure of coverage may provide a stronger indicator [23]. Also, for diatoms in intertidal mudflats the opposite relationship was found, where the truncation of the power law disappeared under increased grazing pressure [24]. Until now, there has been little direct comparison between the properties of patch sizes for a single snapshot and the long-term trends (over several years) of a vegetation population.

Patch-size distributions may also often be viewed as pure power laws with no truncation term, characterized by a single exponent that determines the patchiness of the spatial pattern. The exponent of a power-law patch-size distribution, also known as the Korcak exponent, *K* is defined given a patch area *A* and the number of patches observed of that size *N*_*A*_, using the relationship

$$N_{A}∼A^{-K}.$$

Power-law patch-size distributions that are key to the Korcak exponent are ubiquitous in nature [25] and a variety of ecological models can reproduce such patterns [26,4]. Moreover, simple models of aggregation dynamics, which focus on the rate at which patches of differing sizes coagulate, can produce patch sizes with power-law distributions and there has been some research to indicate this is a universal phenomenon [27,28]. The Korcak exponent gives a sense of the patchiness of the spatial pattern and has been considered in the context of several ecological processes including: correlating with grazing pressure on a landscape [29]; providing an estimate of patchiness and re-forestation [30] and relating to the cover between two species [31]. Mandelbrot [20], and subsequently Hastings [25] and Sugihara [32] proposed that there should be a linear relationship between the Korcak exponent and the underlying dynamics of the process.

However, although the Korcak exponent and other measures related to persistence can be related for particular processes, in general, there is no standard relationship and each measure is independent [33]. It, therefore, remains an open problem whether such fractal exponents are able to give any insight into the persistence of an ecosystem and where the limitations are.

In this paper, we explore when there is a relationship between the spatial and dynamic persistence of an ecosystem and under what scenarios we should expect this relationship to develop. In particular, we use eelgrass (*Zostera marina*), a key marine species around sheltered coastlines, as motivation and a source of high-quality ecological data. The eelgrass around the Isles of Scilly (located off the southwest tip of Cornwall, UK: 49.9° *N* 6.3° *W*) has the key feature that its temporal dynamics can be assembled from extensive surveys over the past 20 years [34], while its spatial distribution has been assessed from aerial photography [35]. From our understanding of eelgrass dynamics, we develop a simple probabilistic cellular automata (PCA) model of clonal growth of vegetation in the presence of an environmental gradient that limits reproduction; we compare and contrast the findings of this model with the data available for eelgrass.

## 2. Lattice-based simulation with environmental gradient

### 2.1 Model development

In order to understand the factors that determine when a relationship between spatial and dynamic persistence (in terms of rates of return to equilibrium density) occurs, we developed an explicit spatial model that includes both demographic and environmental factors. This allows the study of each of these factors separately in order to determine which contribute to the persistence relationship.

We develop a mechanistic model for the clonal growth of vegetation in the presence of an environmental gradient. This model is formulated to capture the known behaviour of eelgrass, but could be parametrized to match a range of ecosystems with a monoculture as the foundation species. The gradient can be used to determine the boundary between regions where the clonal species can colonize and persist, and regions where it is unable to do so due to restrictions in the environment (wave energy, sea depth, temperature, etc.) For example, eelgrass will only colonize coastal regions where sunlight, nutrients and soft-sediment are sufficient, hence an obvious environmental gradient that would determine eelgrass growth is sea depth [36,37].

We consider a single species of plant that can reproduce by local clonal growth; the plants also experience intra-specific competition (competition for nutrients, light, ground water, etc.) from the extended local environment which impacts on their reproductive potential. Plants are assumed to die at a constant rate. The model is formulated as a PCA [38,39], based upon similar assumptions to the kinetic equation modelling vegetative growth given in [40]. Reproduction due to clonal growth or seeding and intra-specific competition are both governed by spatial kernels (*k*_*B*_ and *k*_*C*_) that determine the strength of each process at a given distance. The model is defined on a square *N*×*N* lattice, where each lattice site can either be occupied (1 for short-hand) or unoccupied (0). For ease of explanation, we conceptually consider a plant to occupy a single site, although this is not necessary for any of the results. The four key functions that determine the dynamics of this species are: *λ*(**x**), which captures the environmental gradient and is purely a function of the location **x**; *k*_*B*_(*d*), which captures the rate at which new plants are produced (on unoccupied sites) at a distance *d* from the plant (a Gaussian function with variance $\sigma_{1}^{2}$); *k*_*C*_(*d*), which measures the degree of competition felt from a plant at distance *d* (another Gaussian function with variance $\sigma_{2}^{2}$), while *k* determines the strength of this competition on reproduction.

Mathematically, this can be written as

2.1a $$P_{\text{x}}(0\to 1)=\lambda (\text{x})\left(\sum_{i:\text{occupied}}k_{B}(∥\text{x}-{\text{o}}_{i}∥)\right)\left(1-k\sum_{i:\text{occupied}}k_{C}(∥\text{x}-{\text{o}}_{i}∥)\right)$$

and

2.1b $$P_{\text{x}}(1\to 0)=\mu ,$$

where **o**_*i*_ is the location of the *i*th occupied site. The environmental component *λ*(**x**) determines how growth is modified by environmental factors, and is purely a function of location **x**. *λ*(**x**) has two components: an environmental gradient in a given direction and a site-specific noise term to reflect random perturbations:

$$\lambda (\text{x})=\gamma \text{l}.\text{x}+\xi \eta_{\text{x}}.$$

Here *ξ* is the strength of noise parameter (*η*_**x**_ are independent Gaussian noise terms with mean zero and variance one), *γ* gives the slope of the environmental gradient and **l** is a unit vector that specifies the direction of the gradient.

### 2.2 Model analysis

Simulations were performed on a 100×100 grid for 6×10^4^ time steps after the system has reached an equilibrium state, where the density of occupied sites fluctuates around a mean value. The dynamic (*k*,*μ*), spatial (*σ*_1_,*σ*_2_) and environmental (*γ*,*ξ*) parameters were varied between simulations and the Korcak exponent and return rate were calculated for each simulation run.

The return rate for the simulation is taken to be the expected rate of change in density around the long-term equilibrium, providing an exact counterpoint to the values calculated from the time-series data. For a given spatial pattern at time *t*, the exact probabilities for the births and deaths at each site can be calculated, and a single stochastic realization of these gives the spatial pattern for the next time step. A general form of the return rate can thus be calculated by summing over the probability of all birth events minus the probability of all death events. The expected rate of change of density is therefore $E[\rho_{i}]=\sum_{i}P_{t,i}(B)-P_{t,i}(D)$, where each site is indexed with *i*. Around the equilibrium point, this equation is linearized such that $E[\rho_{t}]=a+b\rho_{t}$. The return rate is the gradient of this linear equation *b*, which was calculated through linear regression. The system was initialized with the north border, where the environmental gradient is at its maximum, fully occupied. This border was fixed in order to ensure stochastic fade-out did not occur. Simulations were run until the population reaches statistical equilibrium, this is assessed as equilibrating of the population density. Once it has reached statistical equilibrium, the density and expected change in density is recorded for a given number of generations *N*. Linear regression is then performed on the dataset and the gradient of regression is taken to be the return rate.

For a given spatial model distribution, the Korcak exponent is calculated as follows. The size of each continuous cluster of occupied sites is calculated, producing a distribution of patch sizes. A power-law (Pareto) distribution is fitted to this data, with scaling exponent and minimum patch size, using a maximum-likelihood estimator approach. The minimum patch size is taken to be 1 pixel (0.2×0.2 m), in order to compare the model-generated and field-generated data directly, and the scaling exponent was estimated via a standard maximum-likelihood method [41].

For the first investigation, dynamic and spatial parameters of the model (corresponding to eelgrass behaviour) were fixed and the environmental parameters were varied (*ξ* and *γ*) between 0 and 1. Assuming each grid cell corresponds to a 20×20 cm area, dynamic and spatial parameters can be set that capture the known behaviour of eelgrass. The death probability was kept constant at *μ*=0.2, the spatial growth and competition scales were kept constant at *σ*_1_=0.5, *σ*_2_=1; while both high (*k*=0.8) and low (*k*=0.2) competition factors were investigated.

For the second investigation, the dynamic parameters (*k* and *μ*) were varied between 0 and 1 for fixed environment and spatial parameters (*σ*_1_=0.5,*σ*_2_=1,*γ*=0.5,*ξ*=0.1).

## 3. Field survey and aerial photography of seagrass meadows

The results from the simulation study are directly compared with the spatial and temporal seagrass data. The aim is to find under what circumstances a relationship between the return rate and the Korcak exponent should exist and then determine if such a relationship can be observed in this real system.

### 3.1 Seagrass meadow locations

**Figure 1.**
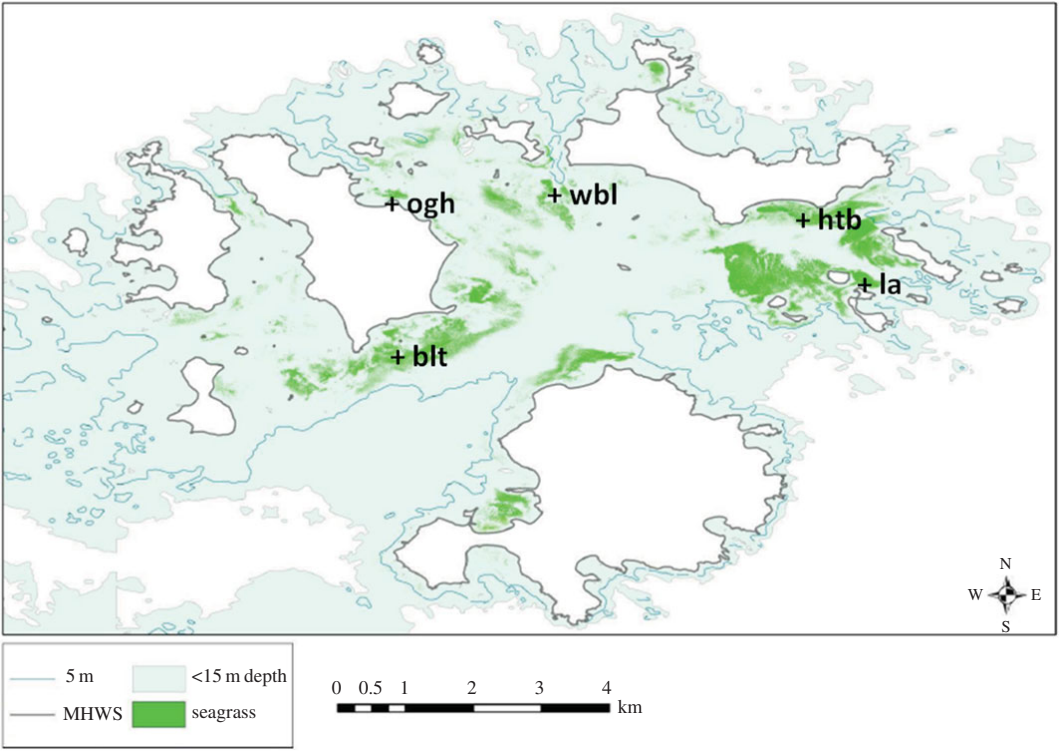
The five surveyed sites in The Isles of Scilly, UK. *blt*,*Broad* Ledges Tresco; *la*,*Little* Arthur; *htb*,*Higher* Town Bay; *ogh*,*Old* Grimsby Harbour; *wbl*,*West* Broad Ledges. Both time-series data in the form of annual surveys and spatial data in the form of a single aerial survey were conducted (adapted from [35]).

We monitored five seagrass (*Z. marina*) meadows around the Isles of Scilly, UK (figure 1 and table 1) from 1996 to 2014, using rigorous and consistent methodology [34,42,43].

**Table 1.** GPS positions and depths relative to chart datum for the five seagrass survey sites (‘+0.5’ indicates this site is exposed at low water, all other sites are fully sub-tidal).

site	latitude and longitude	depth (m)
Broad Ledges Tresco	49°56.4′ N, 06°19.6′ W	0.2
Higher Town Bay	49°57.2′ N, 06°16.6′ W	+0.5
Little Arthur	49°56.9′ N, 06°15.9′ W	1.0
Old Grimsby Harbour	49°57.6′ N, 06°19.8′ W	0.6
West Broad Ledges	49°57.5′ N, 06°18.4′ W	0.6

### 3.2 Survey protocol

Seagrass was surveyed annually, during the first week of August, by placing 25 quadrats (0.25×0.25 ) in each meadow and counting all the shoots visible above the substrate. Quadrat positions were predetermined as random rectangular coordinates (*x*,*y*) translated into polar coordinates (*distance, bearing*), radiating from a chosen focal point. Randomization of quadrat locations was renewed each year and the maximum distance was 30 m from the focal point, close to the centre of each meadow.

### 3.3 Statistical modelling

We developed a metapopulation dynamic model of seagrass habitat occupancy, based on the classic Levins model [44]. Suitable habitat is defined as either occupied or vacant. The proportion of habitat patches for meadow *i*, *N*_*i*_, is dependent on colonization and extinction rates, with dynamics described by a logistic function. In discrete time, this function can follow the standard linearization, such that *y*_*t*,*i*_=*a*_*i*_+*b*_*i*_*N*_*t*,*i*_, where $y_{t,i}=\log(N_{i,t+1}/N_{i,t})$ [45].

In order to calculate the return rate, we estimated habitat occupancy as the proportion of replicate quadrats that were occupied by seagrass each year, *t*, at each of the five survey sites (*i*=1,…,5). In its linearized form, the metapopulation model can be fitted to spatially replicated seagrass data using a generalized additive model (GAM) framework, regressing *y* on *N*, with *a*_*i*_ represented by sitewise *y*-axis intercepts and density dependence described using smoothing splines at the individual site level [46]. The GAM fitting process implements a generalized cross-validation algorithm to assess the optimal degree of nonlinearity. Additionally, we captured differences in within-site temporal variance, as well as between-site correlation, by incorporating a full variance–covariance matrix, estimated directly from the data (see [47] for full details of the method). The regressed value *b*_*i*_ is then taken as the estimate for the return rate at site *i* [17,48].

All analysis was performed using R v. 3.1.2 (R Core Team 2014), with additional functions from the packages: ggmap, ggplot2 and mgcv.

## 4. Results

The spatial-dynamic persistence relationship was compared when either the environmental parameters or the demographic parameters were varied. These relationships were then compared with the seagrass ecosystem, where the dynamic persistence was measured from the annual longitudinal study and the spatial patchiness was measured from the aerial photographic survey described in the methods.

### 4.1 Simulations

**Figure 2.**
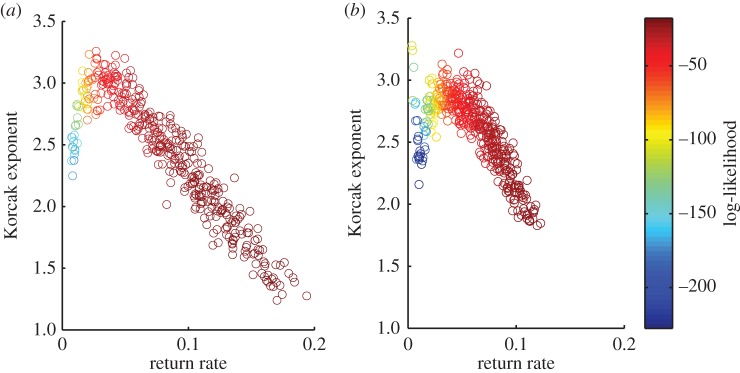
Relationship between return rate and Korcak dimension for high and low competition, where environmental parameters were varied. Other parameters were kept constant at *σ*_1_=0.5,*σ*_2_=1 and *μ*=0.2. The log-likelihood indicates goodness of fit to the spatial data, where higher values indicate a better fit. (*a*) Korcak dimension (low *k*) and (*b*) Korcak dimension (high *k*).

In the initial investigation, the dynamic and spatial parameters of the model were fixed and the environmental parameters were varied. The Korcak exponent correlates strongly with the return rate; there is also a high likelihood for each fitted power-law distribution, indicating a good fit of the exponent (figure 2*a*,*b*). The gradient of the relationship was also found to vary depending on whether high competition or low competition was present.

**Figure 3.**
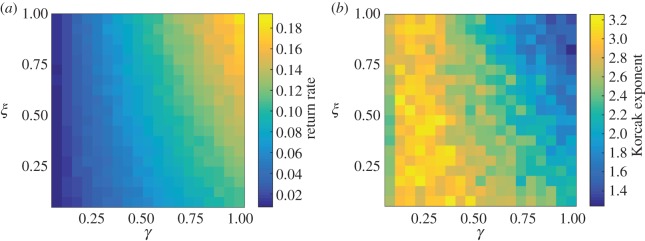
Resulting values of (*a*) return rate and (*b*) Korcak exponent for environmental noise (*ξ*) and environmental gradient (*γ*), where competition (*k*) is low. Both environmental noise and gradient have a large impact on both the static and dynamic properties of the system. A sharper gradient increases the return rate and this effect is more pronounced for higher environmental noise. The gradient term also decreases the patchiness of the vegetation distribution (lower exponent), and this effect again increases for higher values of noise.

The environmental noise and gradient terms both have a large impact on the resulting return rate and Korcak exponent of the system (figure 3*a*,*b*). A higher environmental gradient leads to a higher return rate, with a decreased spatial patchiness (lower Korcak exponent). This effect is also more pronounced when the environmental noise term increases. The return rate and spatial patchiness is at its greatest value, where both the environmental gradient and noise are also at their greatest value.

**Figure 4.**
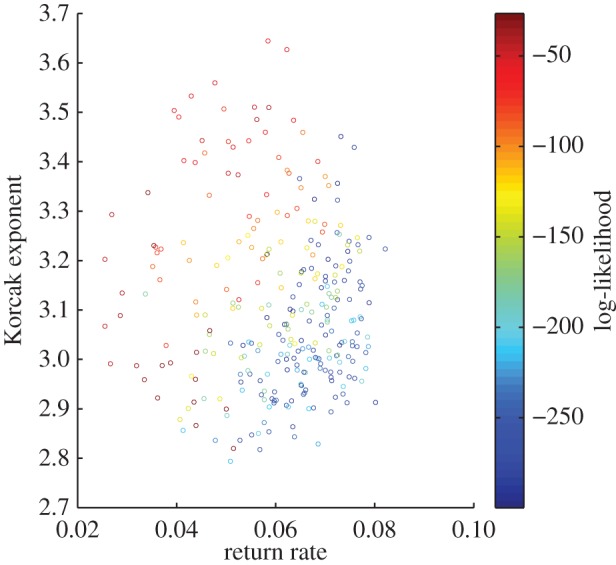
Varying the dynamic parameters *k* and *μ* for fixed spatial and environment parameters *σ*_1_=0.5, *σ*_2_=1, *γ*=0.5 and *ξ*=0.1. There is a lack of strong correlation between the Korcak dimension and the return rate.

In the second simulation experiment, the environmental parameters were fixed and the dynamic and spatial parameters were allowed to vary. The resulting Korcak exponent (figure 4) gives no relationship to the return rate. Although the spatial scaling does vary (between 2.8 and 3.7) there is no clear emergent relationship and a linear regression analysis finds no significant trend (*p*>0.05).

The simulation analysis therefore indicates that a negative-linear relationship is found when sites are near equilibrium and when only environmental variables vary between sites.

### 4.2 Seagrass data analysis

**Figure 5.**
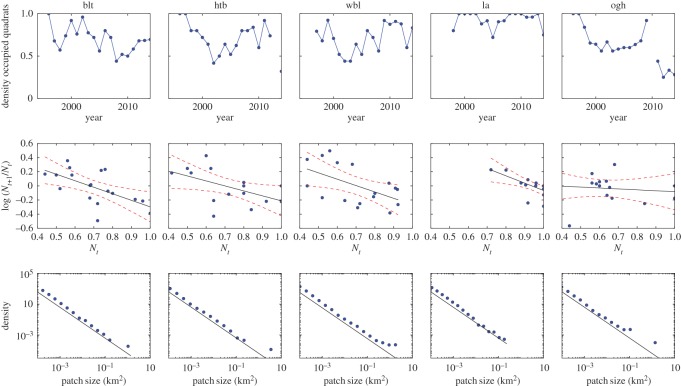
Time-series and patch-size data from five survey sites around the Isles of Scilly, UK (*blt*,*Broad* Ledges Tresco; *htb*,*Higher* Town Bay; wbl, West Broad Ledges; *la*,*Little* Arthur; *ogh*,*Old* Grimsby Harbour). The three sites used in the study are on the left, with the two excluded on the right. *Top row*: Data points represent proportions of occupied 25×25 *cm* quadrats for the years 1996–2014. *Middle row*: Transformed data, comparing density at year *t* to $\log(N_{t+1}/N_{t})$. Regression line for return rate shown in black with 95% confidence limit shown as a red dashed line. *Bottom row*: Patch-size distribution for five sites, with normalized frequency. A solid line with a scaling of 2 has been added for clarity.

Density dependence analysis was undertaken to quantify the return rate of seagrass metapopulations in equilibrium at each survey site. Here, return rate is the negative of the gradient of the density-dependent response around equilibrium [48]. Initially, the fitted variance–covariance matrix was assessed. Inclusion of empirical between-site spatial correlations did improve model fit (Likelihood *ratio*=34.4, *d*.*f*.=10, *p*<0.001). Generalized cross validation showed that a linear functional form, rather than smoothing splines, was the best fit at all sites except Old Grimsby Harbour, making direct estimation of return rates straightforward in all but that case (figure 5). Return rates are shown in table 2. At three survey sites, Broad Ledges Tresco (blt), Higher Town Bay (htb) and West Broad Ledges (wbl), the null hypothesis of random walk dynamics was rejected in favour of density-dependent population regulation [13]. However, at Little Arthur (la) and Old Grimsby Harbour (ogh), statistically significant return rates could not be estimated (figure 5).

**Table 2.** Estimated return rates and Korcak exponent for the five seagrass survey sites. Also given are the standard error (s.e.) of the return rate fit along with the *t*-value and corresponding *p*-value and the 95% CIs for the Korcak exponent estimated through bootstrap re-sampling.

site	return rate	s.e.	*t*-value (*d*.*f*.=50)	*p*-value	Korcak exponent	95% CI
blt	0.857	0.307	2.79	0.007	1.775	(1.773, 1.776)
htb	0.718	0.296	2.33	0.023	1.869	(1.866, 1.869)
la	0.943	0.618	1.52	0.134	1.761	(1.758, 1.762)
ogh	n.a.	n.a.	n.a.	n.a.	1.811	(1.806, 1.813)
wbl	0.930	0.294	3.04	0.004	1.740	(1.735, 1.740)

**Figure 6.**
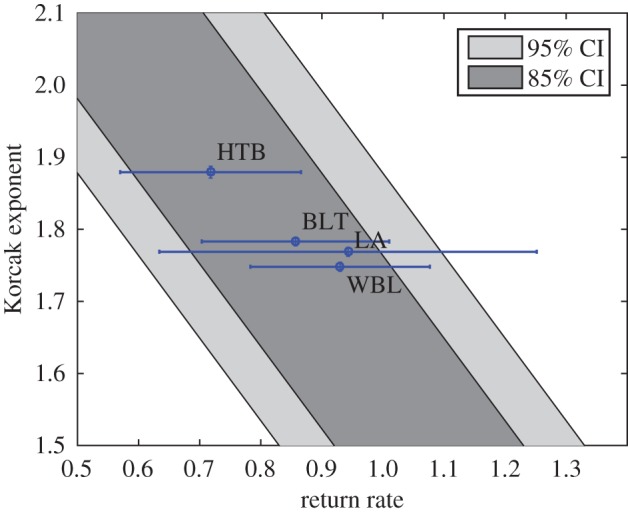
Korcak return rate relationship for surveyed vegetation. Empirical seagrass data for three sites surveyed is shown as labelled points with ±s.e. error bars for both return rate and Korcak exponent, where the Korcak exponent errors were found through bootstrap re-sampling [50]. Although Little Arthur (LA), did not have a statistically significant return rate, this has been plotted here for clarity. The diagonal solid band describes the inverse relationship reproduced by the PCA model over a range of environmental parameters. The overall simulation rate is set using known parameters of death and recruitment for *Zostera marina* [51]. The other simulation parameters were set arbitrarily and hence show a qualitative as opposed to quantitative similarity with the data.

Seagrass persisted at all five survey sites throughout the length of the study (figure 5). There was no evidence of temporal autocorrelation in the time series (Likelihood *ratio*=0.82, *p*=0.37). The only site to show a significant linear trend (decline) was Old Grimsby Harbour (*t*=3.96, *p*<0.001). We found the expected inverse relationship between the power-law exponent of the patch size distribution (the Korcak exponent) and the dynamic return rate, for survey sites that were confirmed as having stationary temporal dynamics (figure 6). However, two sites were excluded as they failed to meet this assumption: Old Grimsby Harbour is evidently in sharp decline (figure 5); and Little Arthur, while maintaining high patch occupancy throughout the survey period, could not be confirmed as being stationary (figure 5 and table 2).

## 5. Discussion

There has recently been increasing interest in finding generic spatial indicators of ecosystem pressure or collapse [22,49]; however, until now there has been little validation of these spatial indicators against long-term population data. We have demonstrated for a particular ecosystem under what circumstances there should be a connection between the dynamic persistence, defined using the return rate to population equilibrium, and the spatial persistence, defined using the Korcak exponent. The simulations derived from a model of a spatial dynamic vegetation system in the presence of demographic competition and an environmental gradient demonstrated that a strong relationship exists only when environmental parameters vary between sites. This relationship was then compared with a long-term seagrass study, where a similar relationship was found for the sites where the return rate could be measured.

We have found that a relationship between the Korcak exponent and the return rate only exists when certain factors are present. Numerical simulation indicates a strong relationship between the Korcak exponent and the rate of return to equilibrium when only properties of the environment are altered. This relationship was supported to a limited extent by data obtained from the long-term study of seagrass. To our knowledge, this is the first time a direct comparison has been possible between time-series and spatial snapshot data on the same natural system. The strategy of coupling rigorous modelling development with high-quality, long-term field study is a powerful approach for making generalizable inference based on biologically well-supported assumptions and observation. In particular, analysis of where the predicted relationship breaks down in nature, where the demographic parameters between locations are significantly different, provides useful motivation and insight for further theoretical exploration.

Using a PCA model, we predict a strong negative linear relationship between the Korcak exponent and the return rate over a wide range of parameters—in fact all parameters that generate return rates above 0.05 (figure 2). This reversal of the relationship can be observed when there is no environmental gradient present (figure 3). While variation in environmental parameters gave a strong Korcak–return rate relationship, this was not duplicated when the dynamic (species-specific) parameters were varied (figure 4). Instead a very low correlation relationship was found. These results highlight the practical usefulness and potential shortcomings of this method for discerning the return rate and hence persistence of a species from its spatial pattern. The change in gradient due to high- and low-competition (*k*) values also gives an indication of how comparisons of the Korcak exponent should be implemented (figure 2). If endogenous parameters (*k*,*σ*_1_,*σ*_2_,*μ*) are fixed then a monotonic relationship is produced with a gradient that is dependent on those endogenous parameters. This suggests that while in some settings the Korcak exponent does correlate with the return rate of the system and thus its persistence, this is not true in general. Other studies have recently highlighted similar results that generic indicators of a catastrophic shift may not hold generally across all settings [19,23,52].

Our results indicate a Korcak–persistence relationship in a long-term study with an aerial photographic survey. Vegetation density data in the form of a 20-year quadrat survey from the Isles of Scilly, UK, was used to estimate the return rate for five distinct ‘meadows’. This was compared with the Korcak exponent measured from the patch-size distribution obtained via an aerial photographic survey conducted towards the end of the study. The same negatively correlated Korcak–persistence relationship was found from this data and has qualitative agreement with the simulation study, subject to the limitations of assuming equilibrium dynamics already noted. The model is simplistic in nature and may not fully capture the variety of complex interactions occurring in a vegetation system, indeed differing model assumptions can lead to different conclusions based on spatial indicators [19,52]. The comparison between the data and model then is given to illustrate the general form of the relationship. A model with more realistic assumptions and fitted parameters may better reproduce the observed relationship therefore. This gives a tantalizing glimpse of how high-resolution remote-sensing techniques could support some more traditional ecological survey techniques when answering questions about the dynamical persistence of an ecosystem. As has recently been pointed out, these spatial techniques would be system-dependent and further validation against empirical data is required before drawing robust conclusions from spatial snapshots alone [52].

In the event, only three out of five sites assessed showed the predicted relationship between spatial and temporal statistics. In both cases where the relationship between spatial and temporal dynamics fell down, it was due to being unable to reliably estimate a return rate from time-series analysis. At one site (Little Arthur), coverage by vegetation was very high for most of the time surveyed. Although population growth rate and density was reasonably described by a linear relationship (figure 5), this presented too little information about response to perturbation to derive a statistically significant return rate. The other site not following the predicted relationship was Old Grimsby Harbour, which is heavily disturbed by boat traffic and can be seen to be declining precipitously (figure 5). These cases point to limitations of classic time-series analysis. It would require further validation of the Korcak exponent approach to confidently use alongside time-series analysis.

Sugihara [32] proposed that dynamics could be inferred from complexity of shape. They illustrated this point, by using a fractional Brownian motion model where the Hurst exponent could be used to detect the persistence of the generating process. Other studies [53] have used this relationship to explore how measures of persistence can relate to dynamics; however, there has been no strong quantitative test of whether the spatial persistence of a landscape relates to the temporal persistence. Here we have tested the generality of this hypothesis by precisely defining dynamic persistence and then relating this to the shape of the resulting distribution. Overall, care must be taken when directly comparing spatial statistics to temporal ones. This general conclusion has also been recently highlighted in the context of comparing truncations in the patch-size distribution to the proximity to a dynamic threshold [22,23]. Universality of fractal growth processes leads to many spatial patterns that are similar in the sense that they share the same scaling relationship [54]. For a fractal measure to be applied in the context of estimating a return rate, we believe that a strong mechanistic understanding of the underlying growth mechanisms is required, which has similarly been found in a study of marine diatoms [24]. We have focused on mechanisms where growth is locally positively correlated with surrounding vegetation and negatively correlated on larger scales. However, there may also be other spatially correlated processes in a vegetative system that lead to changes in the scaling properties of the system such as grazing [22,23,55] or disease [56]. It would be interesting to discern the Korcak–persistence relationship where such other factors are present.

## Data accessibility

The Isles of Scilly dataset including the patch sizes and occupancy for the five meadow sites can be found at: http://dx.doi.org/10.5061/dryad.r95s8.
